# The usefulness of chelation therapy for the remission of symptoms caused by previous treatment with mercury-containing pharmaceuticals: a case report

**DOI:** 10.1186/1757-1626-2-199

**Published:** 2009-11-18

**Authors:** Serafina Corsello, Alessandro Fulgenzi, Daniele Vietti, Maria Elena Ferrero

**Affiliations:** 1 Dipartimento di Morfologia umana e Scienze Biomediche-Città Studi, Università di Milano, Via Mangiagalli, 31, 20133 Milan, Italy

## Abstract

**Introduction:**

A great deal of data regarding the toxicology of mercury has been recently reported. Although the most common human exposures to mercury are currently mercury vapour from amalgam tooth fillings, methylmercury from seafood and ethylmercury as a preservative in vaccines, in the past mercury compounds have been used in the treatment of syphilis.

**Case presentation:**

Mercury intoxication was found in a 67 year-old Italian man affected by neurological symptoms of apparently unknown origin. The patient developed syphilis forty years ago and then underwent therapy with mercurials to treat his chronic bacterial infection. We treated the patient with disodium edetate chelation therapy.

Six months after the beginning of the therapy, the patient's neurological symptoms began to decrease, and were completely cured after two years of therapy.

**Conclusion:**

This case supports the use of chelation therapy with disodium edetate to remove damages caused by mercury intoxication.

## Introduction

Between 1916 and 1955 important advances were made in the treatment of syphilis. During the first years of this period arsenicals, mercurials, malaria therapy and heat therapy were used, then these treatments were progressively replaced by the use of penicillin [1]. However, in the years prior to 1916, the treatment of syphilis consisted of the use of heavy metals, initially with mercurial and later with arsenic and bismuth preparations. Patients treated with heavy metals often developed neurological symptoms which were then described as neurosyphilis [2]. Although new medications supplanted mercury it was still used sporadically for syphilis until the 1960s. The following describes the case of a patient who received mercurial chemotherapy for syphilis forty years ago and developed misunderstood neurological symptoms.

## Case presentation

In September 2004, a 67-year-old Italian man presented to our medical center. In 1964 he was subjected to heavy metal (mercurials) therapy to treat a syphilis infection. The patient remembered that the treatment (which was performed one time a week for 4 months) consisted in 10 ml intravenous injection of a mercurial solution (possibly calomel, e.g. mercurous chloride, Hg_2_Cl_2_, or cinnabar, e.g. mercury sulphide, HgS). Contemporarily, the patient was administered with penicillin (he referred the daily dose of 500,000 units, at alternative months, for 12 months). The patient completely recovered from the disease. However, ten years after (in 1974), he developed some symptoms such as shiver, pallor, asthenia, which lasted for 2 weeks, then disappeared. Such symptoms recurred about any two years. The patient has been examined by a physician, who found hepatomegaly, whereas the blood tests revealed increased azotemia. In 1984, the patient developed convulsions (3 episodes), and 8 years ago he presented headache, tremors, vertigo (often resulting in falling out of bed during the night), memory loss, anxiety, depression, insomnia, muscular cramps, tachycardia. These and previous symptoms were never attributed to any one specific disease by the internists and neurologists who visited the patient over the course of forty years. Only more recently (five years ago) the patient was examined by a physician expert in chelation therapy. The physician, by using the bioresonance method (VEGA test) evidenced the presence of elevated levels of mercury in the patient's body. The physician collected from the patient some samples of urine and analyzed them for mercury. The samples revealed levels of mercury <4 μg/g creatinine, which represent the normal urine reference range of this metal. The same physician provoked the mercury "challenge" of the patient, by treating him with disodium edetate (EDTA) (2 g/10 ml, Salf, Brescia, Italy) diluted in 500 ml physiological saline and intravenously administered by slow infusion (about 90 min) and inviting him to collect urine for 12 hours. After the challenge, the urine mercury levels were 10 μg/g creatinine. The patient has remembered at this time the old mercurial therapy and was addressed by the physician to our centre to perform the chelation therapy. We took the patient's anamnesis, and his past and recent blood tests. On the whole these tests revealed normal levels, with the exception of increased creatinine serum values (1.3 ± 0.7 mg/dL = mean ± SEM, reported in ten different blood tests taken between 1990 and 2004) and a reduced creatinine clearance (63.60 ± 6.2 ml/min).

The inorganic form of mercury, as cinnabar, has been shown to possess neurotoxicological effects, when orally administered in mice [3]. In fact, Huang et al. have demonstrated that mercury of cinnabar (10 mg/kg/day, through oral application by gavage for 11 consecutive weeks) could be absorbed by gastro-intestinal tract and significantly accumulated in cerebral cortex, cerebellar cortex, liver and kidney. Indeed, inorganic mercury is able to pass the blood-brain barrier. A recent work suggest that mercury uptake into hair mimics uptake into brain for both organic and inorganic mercury, e.g. the hair is the best biological indicator of mercury specie levels [4]. Even if the patient was treated with mercurials many years ago, we decided to measure the concentration of heavy metals in his hair samples, having the patient especially neurologic symptoms [3]. We have also measured, before and after the chelation therapy, the oxidative stress profile of the patient, tacking into account that the levels of endogenous antioxidant systems could improve, as compensatory pathophysiological response to the oxidative damage induced by mercury [5]. Indeed, we measured the patient plasmatic levels of the reduced and oxidized forms of glutathione (GSH and GSSG, respectively), and the levels of reactive oxygen species (ROS) (expressed in Carratelli Units or U CARR).

Hair samples were taken from the occipital and temporal regions of the head. Samples were collected with scissors from three different areas of the scalp by cutting 1 cm above hair insertion. About 200 mg of hair samples were obtained, stored in plastic envelopes and transported to the Laboratory of Toxicology (Doctor's Data Inc., St Charles, IL, USA). Hair heavy metal concentrations were determined by Inductively Coupled Plasma-Mass Spectrometry (which guaranteed precision and accuracy of measurements) and were expressed in micrograms per gram (μg/g).

**Figure 1:**
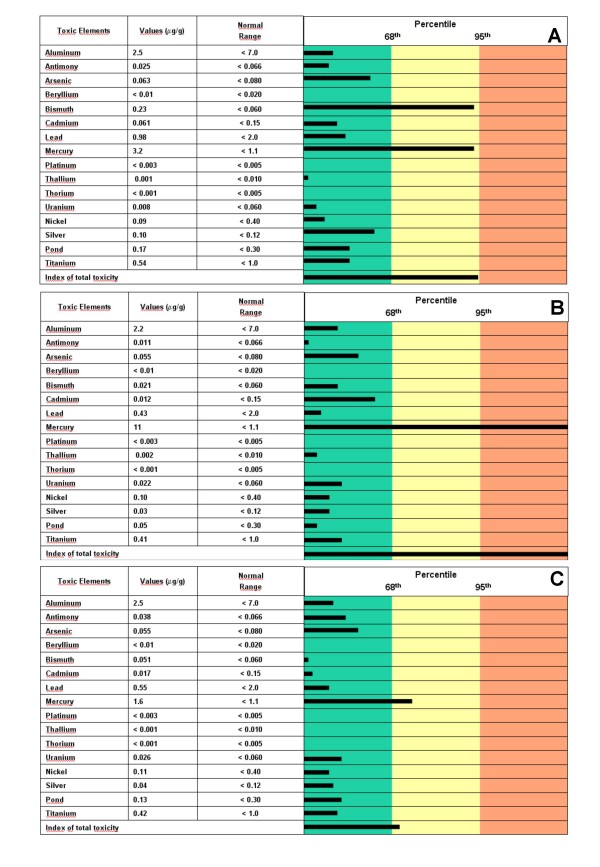
**Temporal heavy metal levels in the hair of the patient**. A = 3 September 2004; B = 7 September 2005; C = 29 September 2006.

The first hair sample was taken on 3 September 2004. It revealed a high content of bismuth (0.23 μg/g: reference values ≤ 0.060 μg/g) and mercury (3.2 μg/g; reference values ≤ 1.1 μg/g) (Figure 1A). The patient was given chelation therapy by intravenous EDTA (2 g in 500 ml saline in about 90 min). The therapy was administered once a week for the duration of a year. We also recommended a diet rich in vitamins and vegetables and invited him to drink large quantities of water. Six months after the beginning of the therapy the patient reported that some of the neurological symptoms had disappeared. He did not fall out of bed at night and had recovered his memory. A year after EDTA chelation therapy was initiated, on 7 September 2005, we carried out the second evaluation of heavy metal levels in the hair (Figure 1B). It showed a very significant increase of mercury levels (11 μg/g) and the normalization of bismuth levels (0.021 μg/g). We continued chelation therapy once a week for another year and on 29 September 2006 carried out the third evaluation of heavy metal levels in the hair (Figure 1C). This evaluation showed mercury levels of 1.6 μg/g. We then administered chelation therapy once monthly to the patient until hair mercury levels were normal. During these periods, the neurological symptoms of the patient (anxiety, depression, insomnia) completely disappeared, his muscular skeletal cramps improved. During the chelation therapy with EDTA we monitored the creatinine levels of the patient once every 90 days. These levels improved after the beginning of chelation therapy over the eight successive evaluations carried out (1.0 ± 0.5 mg/dL), as well as the creatinine clearance values (70.20 ± 5.4 ml/min). Before the beginning of the chelation therapy (which started in September 2004) the patient blood tests showed a GSH/GSSG ratio of 6 and ROS values of 515 U CARR. At the end of chelation (in October 2006) the values were: GSH/GSSG = 13 and ROS = 308 U CARR. At the end of the chelation therapy, we have also measured the urine mercury levels after a 12-h period collection following the EDTA challenge: they were < 4 μg/g creatinine.

## Discussion

The main three forms of mercury exposure today are: 1) inorganic mercury, which includes elemental metallic mercury (Hg^0^) as mercury vapour from amalgam tooth filling, or its mercuric (Hg^++^) oxidation state from food, 2) methyl-mercury (CH_3_Hg^+^) from fish or sea mammalians, 3) ethyl-mercury as preservative thimerosal used in vaccines. In addition, some people are exposed to mercury in occupational settings. The three species of mercury are transported by different mechanisms in the body [6].

**Figure 2:**
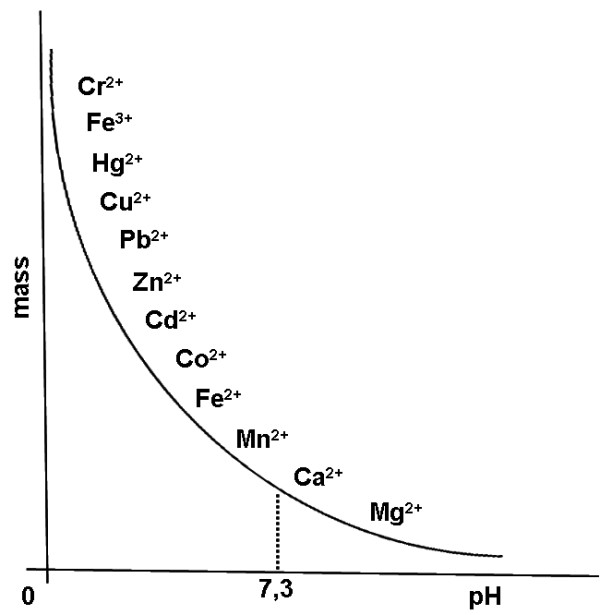
**In vitro affinity of EDTA for metal ions**. The curve represents the affinity of EDTA for different metals, in relation with pH and mass.

We have reported here the case of a patient who was administered with compounds of mercury for the treatment of syphilis many years ago. The patient suffered neurological symptoms for many years which were never attributed to previous mercury treatment. He manifested the neurologic symptoms about 10 years after the therapy with mercurials. Such mercurials were possibly the forms of inorganic mercury known as calomel or cinnabar [7]. We have assumed that inorganic mercury intravenously injected to the patient was then distributed to all tissues through the bloodstream. Hg^2+ ^from inorganic mercury can be biotransformed to Hg^0 ^(in kidney, liver or spleen), then excreted by urine and feces, but Hg^0 ^can be also converted to Hg^2+ ^in brain and kidney [6]. Hg^2+ ^can be bound by EDTA because its high affinity, as reported in the Figure 2. The presence of mercury in the urine's patient after challenge with EDTA as well as the presence of mercury in his hair stimulated us to submit the patient to the chelation therapy.

The chelating drugs used as antidotes to metal toxicity are numerous [8]. Among them EDTA, which is able to bind to many divalent ions with a different affinity, has been used successfully to treat lead intoxication [9,10]. The selection of EDTA for the treatment of mercury poisoning has been justified by the following arguments: i) we necessitated to possibly select a "long-term" chelating treatment, since the mercury poisoning of the patient occurred many years ago. The chelating agents proposed for treating human cases of mercury poisoning, e.g. DMPS (dimercaptopropane-1-sulphonate acid), DMSA (dimercaptosuccinic acid), DPA (D-penicillamine) or NAPA (N-acetyl-D-penicillamine), are responsible for many side effects and are not used for long time; ii) the treatment with these chelating agents is preferably performed orally and not intravenously; we found that only the intravenously administered chelating agent was able to reach the organs and to bind and remove the mercury; iii) we tested the in vivo chelating activity of EDTA and we showed that, by using the "challenge" technique, the urine levels of all toxic metals present in the body's patient were increased (not shown personal data). Previously is has been shown that EDTA use improved renal function and slowed down the progression of renal insufficiency in non-diabetic patients exposed to environmental lead [11]. EDTA in vitro performs most stable complexes with both Pb^++ ^and Hg^++ ^(Figure 2). EDTA treatment is the only chelating agent successfully in vivo intravenously used over a four-year period without side effects in non-diabetic chronic kidney diseases [12].

The first hair sample (Figure 1A) of our patient revealed an elevated content of mercury. Did such levels reflect the old contamination of the patient by mercury-containing drugs? We think that such levels could reflect the dietary habits of the patient, who in the later 5 years was eating seafood for three days a week. However, the second hair sample (Figure 1B) revealed a significant increase of mercury levels one year after chelation therapy. During this year the patient avoided eating fish and sea mammals, following our suggestions, to avoid further sources of mercury. We hypothesize that long term EDTA intravenous treatment was able to remove mercury from the tissues and to favour its elimination through the kidneys and hair. The third hair sample (Figure 1C), obtained after an additional year of treatment with EDTA and characterized by a significant reduction of mercury levels, showed that the removal of mercury from the tissues was nearly complete.

This is the first study reporting the beneficial effects of EDTA chelating therapy to remove an old mercury intoxication. The intravenously injected inorganic mercury reaches the brain via blood circulation. The mercury deposition in the brain provokes many irreversible neurological features, whose improvement relies on the removal of mercury. The neurologic symptoms were not due to the syphilis itself, because the patient, following the treatment with mercurials and penicillin, completely recovered from the disease and did not develop the third stage of the disease, which is the neurosyphilis. Indeed, the neurologic symptoms of the patient were attributable to the mercurial treatment. Such symptoms were different from those due to known neurologic disorders. We have shown here that long term treatment with EDTA was able to cure neurological symptoms in a patient who was administered inorganic mercury to treat a chronic bacterial infection. During such therapy the patient was day by day improved in the symptoms. Chelation therapy with EDTA did not induce any side effects in the main site of EDTA elimination at the time, e.g. the kidneys, as shown by the improved creatinine levels during chelation treatment. Our previous study also demonstrated that the functions of rats kidneys affected by ischemic injury were improved by EDTA treatment [13]. The patient's neurologic symptom disappeared following the end of the therapy until today. The improvement of ROS and glutathione blood levels by the chelation therapy and the absence of mercury levels in urine following the last EDTA treatment confirm our suggestions.

## Conclusion

The reported clinical case should encourage the use of EDTA chelation therapy in patients who suffer from mercury intoxication.

## Abbreviations

EDTA: disodium edetate.

## Consent

Written informed consent was obtained from the patient for publication of this case report and accompanying images. A copy of the written consent is available for review by the Editor-in-Chief of this journal.

## Competing interests

The authors declare that they have no competing interests.

## Authors' contributions

SC was the physician who examined the patient and evidenced mercury intoxication. AF and DV collected the biochemical and clinical data. MEF managed the patient and wrote the paper. All authors have read and approved the final manuscript.
